# The Family‐Centred Practices Scale: Psychometric properties of the Spanish version for use with families with children with Down syndrome receiving early childhood intervention

**DOI:** 10.1111/cch.12970

**Published:** 2022-01-30

**Authors:** María Auxiliadora Robles‐Bello, David Sánchez‐Teruel

**Affiliations:** ^1^ Psychology Department University of Jaen Jaen Spain; ^2^ Faculty of Psychology University of Granada Granda Spain; ^3^ Spanish Society of Clinical and Health Psychology Madrid Spain

**Keywords:** Down syndrome, early childhood intervention, family‐centred practices, psychometric properties

## Abstract

**Background:**

The Family‐Centred Practices Scale (FCPS) assesses the degree to which staff in early childhood intervention and development centres use this therapeutic approach. However, there is no adaptation of this scale to families of children with Down syndrome, which is one of the most prevalent intellectual disabilities in early intervention.

**Objectives:**

To validate and analyse the psychometric properties of the FCPS in Spanish parents with children with Down syndrome receiving early childhood intervention.

**Methods:**

Descriptive analyses, exploratory factor analysis (*n* = 131), confirmatory factor analysis (*n* = 126) and scale reliability analyses were performed. In addition, the invariance of the scale by parents' age and gender was assessed, and a longitudinal analysis of the scores was performed.

**Results:**

A new scale was obtained with a two‐factor structure, similar to the original version, but with fewer items. Goodness‐of‐fit indices were excellent (root mean square error of approximation [RMSEA] [95% confidence interval {CI}] = 0.02 [0.01; 0.04]; comparative fit index [CFI] = 0.98; Tucker–Lewis index [TLI] = 0.97; root mean residual [RMR] = 0.02; goodness‐of‐fit index [GFI] = 0.91; adjusted GFI [AGFI] = 0.90). However, the measure was not gender invariant. Additionally, internal consistency of the two dimensions showed high values in this sample, and comparing the means between the two measurement time points (initial and at about 6 months) showed no differences; the test was powerful but had a very small effect size.

**Conclusions:**

The psychometric properties of this FCPS are adequate, and it uses fewer items, which makes it faster to apply and gives it better clinical applicability. This new version of the scale is a valid, reliable tool for evaluating family‐centred practices in Spanish families with children with Down syndrome.

**Key Messages**

- The Family‐Centred Practices Scale (FCPS) assesses the degree to which staff in early childhood intervention and development centres use this therapeutic approach.
- There is no adaptation of this scale to families of children with Down syndrome.
- The psychometric properties of this FCPS are adequate, and it uses fewer items, which makes it faster to apply and gives it better clinical applicability.
- The two‐dimensional (relational and participatory practices) 10‐item FCPS demonstrates better psychometric properties for this sample.

## 1 INTRODUCTION

In Spain, when a child is born with a disability, or at risk of suffering a disability, they are referred to an early childhood intervention (ECI) and development centre (Robles‐Bello & Sánchez‐Teruel, 2013). These centres target children aged 0–6 years old and their families in order to improve their psychological development and their future adaptation to school (Robles‐Bello et al., 2018; Robles‐Bello et al., 2020). Down syndrome (DS) is a genetic disorder that is accompanied by intellectual disability and particular problems in certain social and adaptation skills (Schalock et al., 2019). Although in Spain the incidence of this syndrome has fallen drastically (de Graaf et al., 2021; Spanish Collaborative Study of Congenital Malformations [SCSCM], 2021), it is still the most frequent form of intellectual disability in the world (Lee et al., 2021; Robles‐Bello & Sánchez‐Teruel, 2019).

According to the American Association of Intellectual and Developmental Disabilities (AAIDD) (2011), the definition of intellectual disability is not based exclusively on limitations but should be based on enhancing capacities and supports that strengthen the psychosocial development of the person with a disability. Specifically, it is defined as “an individual state that is characterized by significant limitations in both intellectual functioning and adaptive behavior, expressed in conceptual, social and practical adaptive skills. This disability originates before the age of 18” (p. 1). Supports are offered from different social environments, with the family being very important (Escorcia et al., 2019; Trivette et al., 2010). Therefore, professionals working with these families should help them to foster their children's abilities in order to enhance their learning (Sánchez‐Teruel & Robles‐Bello, 2015). Practices should be promoted to detect possible family needs and strengths, practices aimed at identifying the supports families have at any given moment and promoting their social and personal resources.

In this regard, there are two clearly differentiated factors of family‐centred principles, namely, relationship‐centred practices and participation‐centred practices (Mas et al., 2018; Trivette et al., 2010). Relationship‐centred practices are those aimed at appropriate clinical actions and include behaviours typically associated with effective helping (active listening, compassion or empathy) and positive staff attributions about the capabilities of programme participants. These types of practices are typically described in terms of behaviours that strengthen interpersonal relationships between programme participants and staff (mutual trust or collaboration) and include parental assessment of the practitioner's beliefs about the family's strengths. Participatory practices include staff actions that engage the family and involve them in choice and decision making. Such practices reinforce existing competencies and provide opportunities to learn new skills (Dunst & Trivette, 2009a).

This therapeutic approach, called family‐centred planning (FCP), serves to promote child development (Mas et al., 2018). According to Escorcia et al. (2019), FCP emphasizes the child's right to fully enjoy their family and social life, so that thanks to appropriate supports, they can have equal opportunities in their family and community environment. In addition, this approach includes actions to facilitate communication with families and guide their experience as parents, helping them to make informed, conscious decisions about their child's intervention process (Valencia & Robles‐Bello, 2020). In particular, when parents have coping skills that focus on the problem rather than the emotion, stress decreases, and family well‐being increases (Masefield et al., 2020). This maximizes the effect of the intervention on the child and promotes a better therapeutic prognosis (Mas et al., 2019).

There are tests for evaluating the effects of FCP (Dunst & Trivette, 2003; King et al., 2004); however, some of them have significant limitations. The *Measure of Processes of Care* (MPOC‐20) is an instrument with 20 items; the reliability of which has not been evaluated nor has its measurement invariance (King et al., 2004). In contrast, the *Family‐Centred Practices Scale*‐*FCPS* (Dunst & Trivette, 2003) seems to be a suitable instrument for measuring professional practices that result from the family‐centred model (Mas et al., 2018). However, the type of disability that the child has can improve or worsen the prognosis of an intervention based on FCP (Goedeke et al., 2019), especially when dealing with children aged between 0 and 6 years old (Ashworth et al., 2019; Mori et al., 2018).

Mas et al. (2018) examined the psychometric properties of the *Family‐Centred Practices Scale*‐*FCPS* for early treatment, although only 14 parents of children with intellectual disability participated in the study, so there was no differentiation between specific populations, such as those with DS. In addition, a subsequent validation in the Spanish population reported significant differences in the numbers of mothers and fathers, with around 80% more mothers than fathers taking part (Mas et al., 2019). A similar limitation had already been noted by the English‐speaking authors of the original (Dunst & Trivette, 2003). The variability of a self‐reported measure with respect to gender is an essential methodological element for the items of an instrument to be understood in the same way in both sexes (Muñiz et al., 2013) and also for instruments to be valid, especially in clinical populations (Hernández et al., 2020). To date, there seem to be no studies that have adapted the *Family‐Centred Practices Scale‐FCPS* and considered measurement invariance depending on whether it is the mother or father doing the reporting, nor have we found validated studies in Spanish parents of children with DS.

For this reason, the present study aims to assess the psychometric properties of the FCPS in a sample of Spanish families with children with DS. More specifically, we aim to analyse the structure and internal consistency of the scale, as well as to determine invariance between genders (mothers and fathers) and longitudinally compare scores after 6 months.

## 2 METHOD

### 2.1 Participants

We first contacted 281 families with children with DS in early childhood care. The parents completed an ad hoc Likert‐type scale with five response options (1 = *never*, 5 = *always*) reflecting the extent to which the intervention programme in the centre was aimed at the family or only at the child (Mas et al., 2018). The inclusion criteria were as follows: (a) having a child with DS, (b) regularly attending the centre for at least 6 months and (c) the child's intervention model in the ECI centre being based on FCP. From the initial total, 24 parents were excluded for not meeting one or more of the inclusion criteria. Ultimately, 257 parents with children with DS from various provinces in the south of Spain participated. Slightly more than half of the respondents (138) were women, 119 were men, and ages ranged from 22 to 46 years old (*M* = 36.5; *SD* = .90). Table 1 gives the most important socio‐demographic variables.

**TABLE 1:** Socio‐demographic characteristics of the families

	*N* (%)	*n*_1_ (%)	*n*_2_ (%)
Gender			
Female	138 (53.70)	72 (57.14)	66 (52.38)
Male	119 (46.3)	59 (42.86)	60 (47.62)
Mean age (standard deviation)	37 (.59)	36.5 (.90)	37.1 (.78)
Civil status			
Single	1 (0.39)	1 (0.77)	0 (0)
Married or in stable partnership	235 (91.44)	120 (91.60)	115 (91.27)
Separated/divorced (living alone)	6 (2.33)	4 (3.05)	2 (1.59)
Separated/divorced (with partner)	15 (5.84)	6 (4.58)	9 (7.14)
Educational qualifications			
No qualifications	12 (4.66)	4 (3.06)	8 (6.35)
Primary education	68 (26.46)	38 (29.01)	30 (23.81)
Secondary education	94 (36.58)	49 (37.40)	45 (35.71)
University or higher	83 (32.30)	40 (30.53)	43 (34.13)
Employment			
Full time	170 (66.15)	80 (61.07)	90 (71.43)
Part time	38 (14.79)	23 (17.56)	15 (11.90)
Unemployed	49 (19.06)	19 (21.37)	30 (16.67)
Mean number of months attending ECI (time)	30.4	28.7	31.2
Total	257	131	126

Abbreviation: ECI, early childhood intervention.

### 2.2 Instruments

Socio‐demographic data sheet: This collected the respondent's sex, age, who was completing the instrument, civil status, educational qualifications, employment, and time spent attending early intervention.

The Family‐Centred Practices Scale‐FCPS (Dunst & Trivette, 2003), translated into Spanish by Mas et al. (2018): This is a self‐report for evaluating how much the staff at the ECI centres use FCP‐based methodology. The original scale has 12 items and two subscales, one with six items which measures the relationships between parents and staff, called *relational practices* (RPs), and the other, also with six items, that includes aspects related to parental participation encouraged by staff, called *participative practices* (PPs). RPs are those aimed at achieving appropriate clinical activities such as active listening and empathy and include the evaluation of the staffs' beliefs about the strengths of the family. PPs are aimed at understanding the family's concerns, needs and priorities, including decision making and achieving set targets (Dunst & Trivette, 2009b). The responses were given on a 5‐point Likert‐type scale from 1 = *never* to 5 = *always*. The original scale produced an alpha coefficient of .93 (*α*_RP_ = .91 and *α*_PP_ = .91) (Dunst & Trivette, 2003), whereas the Spanish version gave an overall alpha coefficient of .91 (*α*_RP_ = .81 and *α*_PP_ = .83) (Mas et al., 2018).

### 2.3 Procedure

First, we contacted the authors of the Spanish version of the FCPS to obtain the original scale and to inform them of our study (Mas et al., 2018). Subsequently, approval was obtained from the ethics committee at the University of Spain (Code: ABR.20/5.TFM). Meanwhile, we contacted the presidents and directors of various DS associations with ECI centres in the south of Spain. All of the staff were informed about the research project orally and in writing, and ultimately the study was performed in three centres. Following approval from the centres, they provided us with contact details for the parents, to whom we sent a document explaining the study and its main objectives. Subsequently, we contacted the parents who volunteered to participate and gave them more detailed information about the process, as well as assuring them of the confidentiality of their personal data. Parents were evaluated separately, with each parent being given an FPCS questionnaire and the socio‐demographic data sheet individually, in order to consider both parents' experiences in ECI.

Data collection began in January 2020 without interference in the therapeutic process and without interrupting families' planned sessions. The questionnaires were given to the parents to complete between sessions, and they sent the information back the following week by email. Finally, we performed a follow up, repeating the family evaluations at 6 months by administering the scale again, giving us meaningful longitudinal results about the FCPS. All longitudinal analyses of mothers and fathers were matched. On 15 March 2020, the entire Spanish population went into compulsory confinement to the home in response to the COVID‐19 pandemic (Boletín Oficial de Estado‐BOE, 2020). Parents continued to carry out the early care sessions online throughout this period. The situation affected the second data collection at 6 months, although during June 2020, the health situation in the country relaxed considerably, so that the questionnaires completed by families could be accessed via email or through centre staff.

### 2.4 Data analysis

This study uses an ex post facto descriptive design. The data obtained were analysed by factor analysis, reliability analysis and model invariance by gender and age. The analyses were done using the statistical program packages SPSS version 20 and FACTOR 10 (Lorenzo‐Seva & Ferrando, 2006). Based on the parameters proposed by the authors, explained in the section that describes how the instrument is used, confirmatory factor analysis (CFA) was done using the SPSS 23 AMOS (IBM Corporation, 2013) estimation method (Kline, 2016).

## 3 RESULTS

### 3.1 Descriptive analysis of the items (*n* = 257)

In general, the data from the item and internal consistency analyses indicated notable variability in asymmetry and kurtosis in this sample (Table 2), which is indicative of a lack of univariate normality. We found some problematic items in RPs, specifically Items 2, 4 and 5, where the correlation with the total score was very low (<.50), and the overall alpha value for the test (*α* = .57) increased when those items were removed.

**TABLE 2:** Descriptive statistics, indices of asymmetry and kurtosis and item analysis

FCPS‐Spanish	*M* (*SD*)	K‐S	S	K	*r* item total	*α* item removed
			*SE* (−1.06)	*SE* (2.69)		
Relational practice (RP) subdimension						
1	3.89 (0.43)	0.90**	0.17	0.72	0.67	0.45
2	2.76 (0.88)	0.81**	0.09	−0.81	0.21	0.87
3	3.91 (0.56)	0.86**	0.07	−0.78	0.72	0.52
4	4.19 (0.68)	0.86**	−0.05	1.01	0.31	0.48
5	2.15 (1.05)	0.80**	0.03	−0.77	0.02	0.81
6	3.85 (0.61)	0.80**	0.05	−0.86	0.51	0.52
Participative practice (PP) subdimension						
1	4.2 (1.99)	0.97**	0.06	−0.78	0.55	0.53
2	4.6 (0.47)	0.89**	−0.09	−0.92	0.71	0.50
3	4.13 (0.68)	0.90**	0.13	−0.88	0.65	0.49
4	4.28 (0.43)	0.86**	0.10	−0.81	0.52	0.51
5	4.58 (0.57)	0.70**	−0.37	−0.26	0.59	0.53
6	4.61 (0.71)	0.83**	0.49	−0.61	0.72	0.46
Total	72.26 (7.77)	0.98**	0.05	1.68	1	0.57

Abbreviations: K, kurtosis; K‐S, Kolmogorov–Smirnov test; *M*, Mean; S, asymmetry; *SD*, standard deviation; SE, standard error of asymmetry and kurtosis.

* Significant correlation at 0.05 (bilateral).

** Significant correlation at 0.01 (bilateral).

### 3.2 Exploratory factor analysis (*n*_1_ = 131)

The Kaiser–Meyer–Olkin (KMO = 0.81) sampling adequacy index, Bartlett's sphericity test (*χ* ^2^ = 20019.2; *p* < .001), and the determinant of the correlation matrix (.005) demonstrated the suitability of the data for exploratory factor analysis (EFA) (Nunnally & Bernstein, 1995). The FACTOR program compares the mean or the 95th percentile of the factor's percentage of common variance explained from the randomly permutated data to the observed explained common variance from the sample. If the observed percentage of a factor is greater than the random percentage, the factor is retained. This happened twice with the FCPS‐Spanish. Therefore, we extracted two dimensions that explained 29.63% (Factor I) and 29.93% (Factor II) of the variance (based on eigenvalues) (Table 3). As the table shows, the factorial loading for each item was over .50 in each dimension. However, there were complex items that loaded in both subdimensions or that did not load on their theoretical dimension (Items 3 and 5). This led us to decide to perform the confirmatory analysis for the overall scale (Model 1) and remove the complex items (Model 2).

**TABLE 3:** Exploratory factor analysis for FCPS (*n*_1_ = 131)

	Dimensions		
	Factor 1	Factor 2	*h* ^2^
RP			
1	**0.53**	0.11	0.58
2	**0.68**	0.05	0.43
3	**0.61**	**0.55**	0.37
4	**0.65**	0.40	0.39
5	0.38	**0.57**	0.28
6	**0.59**	0.06	0.53
PP			
7	0.17	**0.63**	0.46
8	0.19	**0.62**	0.19
9	0.10	**0.79**	0.12
10	0.02	**0.52**	0.52
11	0.01	**0.59**	0.22
12	0.13	**0.56**	0.14
% variance	29.63%	29.93%	

*Note*: Rotated loading with values >.50 in bold.

Abbreviations: Factor 1 (RP), relational practices; Factor 2 (PP), participative practice; *h* ^2^, Communities.

### 3.3 Confirmatory factor analysis (*n*_2_ = 126)

The results of the analysis of multivariate normality in the second sample (*n*_2_ = 126) showed that there was no multivariate normality in the distribution of the items (Mardia = 437.51) (Mardia, 1970). The results in Table 4 show that Model 2, removing the problematic Items 3 and 5, produced very good goodness of fit indices for the FCPS in this sample. In Model 2, we observed a suitable, significant *χ* ^2^/*df*. All of the other indices were excellent; root mean square error of approximation (RMSEA) (95% confidence interval [CI]) below .06, appropriate values for comparative fit index (CFI) and Tucker–Lewis index (TLI), and goodness‐of‐fit index (GFI) and adjusted GFI(AGFI) above the 0.85 limit, with good agreement between the GFIs. Based on these results, the fit and suitability of Model 2 was considered to be good.

**TABLE 4:** Goodness of fit indices for the confirmatory factor analysis (CFA) for FCPS (*n*_2_ = 126)

	*χ* ^2^	*df*	*χ* ^2^/*df*	*p*	RMSEA (95% CI)	CFI	TLI	RMR	GFI	AGFI
Model 1	122.11	46	4.32	0.00	0.07 (0.03; 0.09)	0.88	0.77	0.08	0.81	0.84
Model 2	34.45	22	2.14	0.00	0.02 (0.01; 0.04)	0.98	0.97	0.02	0.91	0.90

*Note*: Model 1, confirmatory factor analysis for the overall scale; Model 2, confirmatory factor analysis with complex items (3 and 5) removed.

Abbreviations: AGFI, adjusted goodness‐of‐fit index; CFI, comparative fit index; *df*, degrees of freedom; *p*, significance level; RMR, root mean residual; RMSEA, root mean square error of approximation; TLI, Tucker–Lewis index; *χ* ^2^, chi square; *χ* ^2^/*df*, chi‐square goodness‐of‐fit index.

### 3.4 Invariance of the measure (*n*_2_ = 126)

The CFA models added for gender (fathers and mothers) exhibited good fit to the data, indicating that a multiple group CFA was appropriate. However, the configural invariance test for gender (reference model) demonstrated problems in variability. We found that complete scalar invariance was adequate (ΔCFI = .004) but that metric invariance was not (Δ*χ* ^2^_[9]_ = 41.12; *p* > .05; ΔCFI = .08), indicating that the factors did not load equally in men and women. These results suggest that there was no invariance in the measure by gender in this sample (Table 5).

**TABLE 5:** Indices of fit for tests of measurement invariance by gender (fathers and mothers)

	*χ* ^2^	*df*	*χ* ^2^/*df*	*p*	RMSEA (95% CI)	CFI	Δ*χ* ^2^	ΔCFI	
Men (*n* = 60)	38.22	21	1.85	0.05	0.01 (0.01; 0.03)	0.92			
Women (*n* = 66)	39.47	21	1.54	0.00	0.02 (0.01; 0.03)	0.97			
Configural invariance	88.54	70	2.01	0.32	0.03 (0.02; 0.03)	0.98			
Scalar invariance	187.65	71	1.89	0.43	0.01 (0.01; 0.03)	0.95	27.53^ns^	0.004	
Metric invariance	165.12	72	2.98	0.00	0.04 (0.02; 0.08)	0.91	41.12**	0.08	

Abbreviations: CFI, comparative fit index; *df*, degrees of freedom, ns = not significant; *p*, significance level; RMSEA, root mean square error of approximation; ΔCFI, test of difference between comparative fit indices; Δ*χ* ^2^, test of difference between the metric and scalar invariance models; *χ* ^2^, chi square; *χ* ^2^/*df*, chi‐square goodness‐of‐fit index.

* *p* < .05.

** *p* < .01.

### 3.5 Reliability and comparison of means (longitudinal)

Table 6 indicates the consistency of the results via the Cronbach alpha coefficient and the omega coefficient, with values for the overall scale and for the two subscales being adequate. The total alpha for the FCPS (Appendix A) was .91, which indicates excellent internal consistency, and for both coefficients, the indices of consistency were acceptable. Comparison of the means between the two testing time points (initial and after approximately 6 months) did not demonstrate differences between the two; there was notable test power, but a very small effect size.

**TABLE 6:** Descriptive statistics, reliability, comparison of means, test power, and effect size (*n*_2_ = 126)

	*M* (DT)	Min.	Max.	K‐S	A(.32)	K (0.63)	*ω*	*α*	*t*	*η* ^2^	Pow.
FCPS	72.09(3.10)	40	120	0.16**	−1.11	2.31	.89	.91	15.23^ns^	0.02	1.01
RP	3.91(2.32)	2.65	5	0.54**	0.27	0.64	.82	.86	11.14^ns^	0.03	0.98
PP	3.70(1.27)	1.69	4	0.19**	−1.26	1.29	.83	.79	10.23^ns^	0.05	0.91

Abbreviations: A, asymmetry; FCPS‐DS, Family‐Centred Practice Scale for Spanish parents of children with Down syndrome; K, kurtosis; K‐S, Kolmogorov–Smirnov test; *M*, mean; Max, maximum; Min, minimum; *p* < 0.01; Pow., Power of the test; *SD*, standard deviation of error of asymmetry and kurtosis; *t*, test statistic at 6 months; *α*, Cronbach alpha; *η* ^2^, eta squared; *ω*, omega coefficient.

## 4 DISCUSSION

This study aimed to assess the psychometric properties of the Spanish version of the Family‐Centred Practices Scale by Mas et al. (2018) on a sample of parents of children with DS who were receiving ECI. We analysed the structure and internal consistency of the scale and tested measurement invariance between genders (mothers and fathers), as well as comparing scores longitudinally at 6 months.

Research about FCP has shown that the approach is effective in improving interactions between parents and children and the development of children with DS (Masefield et al., 2020). To some extent, it is connected to the concept of family, in which Bronfenbrenner (1974) noted the importance of the way support and resources within the reach of the family were used in order for them to be effective within the family. Staff use of FCP is related to family well‐being and quality of life, as well as to the staff gaining the knowledge, skills and abilities they need to achieve interdisciplinary collaboration when dealing with children who attend an ECI centre (Mas et al., 2019). Parents learn to modify certain beliefs and behaviours to influence their children's learning; they become more aware of their active role in this process of continual development and can improve their family dynamics and quality of life in general (Robles‐Bello et al., 2020; Valencia & Robles‐Bello, 2020).

EFA suggested that the two‐dimensional structure of the FCPS was similar to the original version. However, two complex items were found that loaded on both dimensions. Specifically, Item 3 (“staff understand my child's and family's situation”) and Item 5 (“staff do what they say they will do”) theoretically correspond to the RPs dimension. Further structural analysis confirmed a two‐dimensional structure with fewer items in this dimension. The finding that there are items that do not load adequately on the factor on which they should originally load and the confirmation of a better fit of the data to the reduced version of the scale can be interpreted as suggesting that this 10‐item version with two dimensions may be relevant to this population of parents of children with DS. In terms of invariance of the measure with respect to gender, the CFA models specified for men and women showed a good fit to the data and a multigroup CFA was appropriate. However, the non‐invariance of the measure suggests that fathers and mothers understand the FCPS items differently, which should be considered when applying it to both parents. Similar results with respect to gender have been found in the adaptation of this scale to parents of children with autism spectrum disorder (ASD), although in that case, there were no complex items with factor loadings on both dimensions (Robles‐Bello & Sánchez‐Teruel, 2021).

Parental stress has been identified associated with various disabilities such as ASD, DS and other syndromes (Woodman et al., 2015), so it may be that each family's stress level and well‐being is modulated differently depending on the child's disability or disorder (Mori et al., 2018). This suggests several issues of interest. First, early intervention for children with disabilities should be syndrome specific (Ashworth et al., 2019). Additionally, the pattern of family response to a child's disability seems to be modulated not only by the type of disability but also by the set of resources available (Mas et al., 2019). For example, perceived social support from friends is a determinant in reducing family stress in parents of children with autism (Drogomyretska et al., 2020), but this has not been observed in parents of children with DS (Picardi et al., 2018). One interpretation is that the family environment can enhance or hinder the effectiveness of early intervention, because the help and support needed in families enhances their parental commitment and can at the same time provide them with relational resources to improve their self‐confidence and control over the process, which could ultimately boost early child development.

The behavioural phenotype manifests itself in this population of people with DS differently from other developmental disorders such as ASD. These behavioural phenotypes are a greater probability that people with a certain syndrome exhibit certain behavioural or developmental sequelae, compared with people who do not have it (Hodapp & Dykens, 2005), so there are numerous studies that address aspects related to DS from the specificity of the (Flórez et al., 2015; Ruíz, 2016), for example, emotional intelligence (Sánchez‐Teruel et al., 2020), reading processes (Robles‐Bello et al., 2020), learning potential (Valencia & Robles‐Bello, 2017), etc., have been studied. As far as the family is concerned, Mas et al. (2016) suggest that the more effort involved in raising a child, the more resources the family will need to cope with this situation. From the above, it is possible to identify the greatest support needs in families that show an alteration in relation to typical development and, especially, in those disorders whose functioning profile increases the demands on the family environment, that is, depending on the type of disability of the child. This consideration suggests that the well‐being of the family is influenced by the type of disability of the child. The rarity of the disorder affects families in such a way that they feel they have less professional and social support and less information to assist in the treatment of their children (Dunst & Espe‐Sherwindt, 2016).

Furthermore, parents of children with DS are known to give worse ratings to the attitudes of staff than parents of children with ASD (Federación Estatal de Asociaciones de Profesionales de la Atención Temprana [FEAPAT], 2011). Initial results in families of children with ASD have shown that when assessed via the PCFS, the original two‐factor theory (relational and participatory) is supported, but there seem to be differences in some variables of interest such as gender (Robles‐Bello & Sánchez‐Teruel, 2021), which is a first step towards considering whether or not there are different aspects between parental perceptions depending on the type of disorder children have (DS or ASD) or whether there are other psychosocial variables that may be influencing parents' resources.

There are several limitations to this study. One is that the convenience sampling method we used limits the generalizability of the findings. Another is that the adaptation of the FCPS for a particular population (parents of children with DS) may also affect the generalization of results to samples of parents of children with an intellectual disability other than DS. This aspect should be analysed with a different population to confirm the suitability of this scale. Another important aspect relates to the follow‐up part of the study, as the data may have been affected by the recall effect and may also have been influenced by the increased involvement of parents in the early intervention process at 6 months due to the COVID‐19 pandemic, which led to an increase in online intervention processes. This means that test–retest reliability should be replicated in the future.

### 4.1 Conclusions

The two‐dimensional (relational and participatory practices) 10‐item FCPS (Appendix A) demonstrates better psychometric properties than the original 12‐item scale for the sample in this study. The FCPS was assessed for reliability and validity using a convenience sample of families with children with DS receiving ECIs. To our knowledge, this is the first study that has attempted to assess the validity and reliability of this scale in parents of children with DS, exploring its structural features and confirming the most appropriate structure in this sample. The FCPS as a whole presents good internal consistency and has adequate psychometric properties.

## FUNDING INFORMATION

There is no funding to report for this submission.

## CONFLICT OF INTERESTS

The authors of this article declare no conflicts of interest.

## Data Availability

The data that support the findings of this study are available from the corresponding author upon reasonable request.

## Appendix A FAMILY‐CENTRED PRACTICES SCALE FOR PARENTS OF CHILDREN WITH DOWN SYNDROME FCPS

This scale has been designed to be completed by the parents or main carers of the child with Down syndrome attending early childhood intervention.

**Table:**

		*Never*	*Occasionally*	*Sometimes*	*Usually*	*Always*
	Relational practice (RP) subdimension					
1	The staff really listen to my concerns and requests/Los profesionales realmente escuchan mis preocupaciones o demandas.	1	2	3	4	5
2	The staff see my child and my family in a positive healthy way/Los profesionales ven a mi hijo/a y a mi familia de manera positiva y saludable.	1	2	3	4	5
3	The staff recognize my child's and family's strengths/Los profesionales reconocen las fortalezas de mi hijo/a y mi familia.	1	2	3	4	5
4	The staff recognize the good things I do as a parent/Los profesionales reconocen las cosas buenas que hago como padre/madre.	1	2	3	4	5
	Participative practice (PP) subdimension					
5	The staff provide me with the information I need to be able to make good choices/Los profesionales me proporcionan la información que necesito para poder tomar buenas decisiones.	1	2	3	4	5
6	The staff are responsive to my requests for advice and help/Los profesionales son sensibles a mis peticiones de asesoramiento o ayuda.	1	2	3	4	5
7	The staff help me to be an active part of getting required resources and support/Los profesionales nos ayudan para que participemos de forma activa a la hora de conseguir los recursos y los apoyos que deseamos.	1	2	3	4	5
8	The staff are flexible when my family situation changes/Los profesionales son flexibles cuando mi situación familiar cambia.	1	2	3	4	5
9	The staff help me learn how to do things that benefit my child and family/Los profesionales me ayudan a aprender a hacer cosas que benefician a mi hijo/a y a mi familia.	1	2	3	4	5
10	The staff support me when I make decisions/Los profesionales me apoyan cuando tomo una decisión.	1	2	3	4	5
